# Mitigating Seabird Bycatch during Hauling by Pelagic Longline Vessels

**DOI:** 10.1371/journal.pone.0084499

**Published:** 2014-01-06

**Authors:** Eric Gilman, Milani Chaloupka, Brett Wiedoff, Jeremy Willson

**Affiliations:** 1 College of Natural Sciences, Hawaii Pacific University, Honolulu, Hawaii, United States of America; 2 Ecological Modeling Services, St. Lucia, Queensland, Australia; 3 Pacific Islands Regional Office, National Marine Fisheries Service, Honolulu, Hawaii, United States of America; University of Lleida, Spain

## Abstract

Bycatch in longline fisheries threatens the viability of some seabird populations. The Hawaii longline swordfish fishery reduced seabird captures by an order of magnitude primarily through mitigating bycatch during setting. Now, 75% of captures occur during hauling. We fit observer data to a generalized additive regression model with mixed effects to determine the significance of the effect of various factors on the standardized seabird haul catch rate. Density of albatrosses attending vessels during hauling, leader length and year had largest model effects. The standardized haul catch rate significantly increased with increased albatross density during hauling. The standardized catch rate was significantly higher the longer the leader: shorter leaders place weighted swivels closer to hooks, reducing the likelihood of baited hooks becoming available to surface-scavenging albatrosses. There was a significant linear increasing temporal trend in the standardized catch rate, possibly partly due to an observed increasing temporal trend in the local abundance of albatrosses attending vessels during hauling. Swivel weight, Beaufort scale and season were also significant but smaller model effects. Most (81%) haul captures were on branchlines actively being retrieved. Future haul mitigation research should therefore focus on reducing bird access to hooks as crew coil branchlines, including methods identified here of shorter leaders and heavier swivels, and other potentially effective methods, including faster branchline coiling and shielding the area where hooks becomes accessible. The proportion of Laysan albatross (*Phoebastria immutabilis*) captures that occurred during hauling was significantly, 1.6 times, higher than for black-footed albatrosses (*P. nigripes*), perhaps due to differences in the time of day of foraging and in daytime scavenging competitiveness; mitigating haul bycatch would therefore be a larger benefit to Laysans. Locally, findings identify opportunities to nearly eliminate seabird bycatch. Globally, findings fill a gap in knowledge of methods to mitigate seabird bycatch during pelagic longline hauling.

## Introduction

Ecological objective of ecosystem-based fisheries management include preventing irreparable harm to populations of associated and dependent species and broad, direct and collateral community- and ecosystem-level effects [1]–[4]. Fisheries that target relatively fecund species with r-selected life history characteristics, including pelagic longline fisheries, can cause large impacts on incidentally caught species with K-selected life-history strategies, including seabirds. On the order of hundreds of thousands of seabirds are caught annually in pelagic and demersal longline fisheries worldwide, threatening the viability of some populations of albatrosses, petrels, shearwaters and other seabird species [5]–[8].

Seabirds are primarily caught while longline gear is being set, and as a result, seabird bycatch mitigation research has largely focused on methods to reduce seabird captures during setting and not hauling [9], [10]. There has been substantial progress in identifying effective and commercially viable gear technology solutions to seabird bycatch during setting in pelagic longline fisheries, involving changes in fishing gear and methods. Despite this progress, deficits in fisheries governance systems have largely resulted in nominal progress globally in industry uptake of these best practice mitigation methods [4], [10], [11]. However, the management system for the Hawaii pelagic longline fisheries is an exception [6], [7], [12]–[14].

Since June 2001, when requirements for the Hawaii longline swordfish fishery to employ gear technology methods to mitigate seabird bycatch were introduced, seabird catch rates and levels declined by an order of magnitude. Before these measures, the average nominal annual seabird catch rate was 0.55 seabirds per 1,000 hooks (±0.07 SD of the mean). Following seabird regulations, the average nominal annual seabird catch rate, using data through the end of 2011, was 0.04 seabirds per 1,000 hooks (±0.009 SD of the mean) [7], [12]–[14]. Prior to the seabird regulations, an average of 695 (±216 SD of the mean) annual seabird captures occurred in the fishery (based on data from 1994–2000, catch rate of 0.55 seabirds per 1,000 hooks estimated from ca. 5% observer coverage, and total effort based on logbook data from NMFS [15]). From 2005–2011, when the fishery was subject to seabird regulations and 100% observer coverage, the average annual seabird catch level was 61 (±12 SD of the mean).

The fishery catches primarily Laysan (*Phoebastria immutabilis*) and black-footed (*P. nigripes*) albatrosses: Of 517 seabirds observed captured between 5 May 2004, when a legal definition of a shallow-set came into effect, through 11 October 2012, only two were not a Laysan or black-footed albatross (one sooty shearwater [*Puffinus griseus*] and one Northern fulmar [*Fulmarus glacialis*]). The IUCN Red List categorizes the Laysan albatross as Near Threatened, black-footed albatross as Vulnerable, sooty shearwater as Near Threatened, and Northern fulmar as Least Concern [14], [16]. These four species are not listed as endangered or threatened under the U.S. Endangered Species Act [14].

About 30 vessels are active annually in the Hawaii shallow-set longline fishery, which primarily targets broadbill swordfish (*Xiphias gladius*). The fishery operates year-round, with the majority (>50%) of effort occurring from February through April. Vessels fish at grounds in the North Pacific, north of the Hawaii archipelago, between 130° W to 180° longitude and 22° to 40°N latitude [14], [17], [18].

Under current amended seabird regulations, Hawaii longline swordfish vessels are required to either: (i) Side set, attach weights that are a minimum of 45 g to branch lines within 1 m of the hook, deploy a bird curtain aft of a mainline shooter, and deploy the mainline a minimum of 1 m forward from the stern; or (ii) Night set (setting can be conducted only between the times of one hour after local sunset and one hour before local sunrise); when seabirds are present, discharge fish, offal (fish parts) or spent bait while setting or hauling from the opposite side of the vessel from where gear is being set or hauled; and use only completely thawed, blue-dyed bait [19], [20]. Gilman et al. [7] described these methods. Most swordfish vessels opt to stern and night set [14]. As a result, vessels haul primarily during the daytime when Laysan and black-footed albatrosses most actively forage [21]. Of prescribed seabird bycatch mitigation methods, swivel weight amount, distance between the weight and hook, blue-dyed fish bait, time of day of hauling, and discharging offal, bait and discards of dead and live fish during hauling might significantly affect seabird bycatch during hauling [6], [10].

We analyzed observer data to develop a standardized catch model for live seabirds caught by the Hawaii longline swordfish fishery, enabling the identification of variables that have a significant effect on live seabird captures. Standardized catch rate models, when fit with high quality records from large observer program datasets, enable the identification of variables that significantly affect catch rates, including of species that are vulnerable to unsustainable population-level effects from fishing mortality (reviewed in [13]). Factors were included in the model in an attempt to standardize or account for confounding factors of temporal variability in gear and methods known to significantly affect nominal catch rates (e.g., see [13]). Findings enable the identification of opportunities to further reduce seabird fishing mortality. The hypothesis that seabirds retrieved alive were captured during hauling was tested through a review of observer records.

Study aims were, globally, to fill a gap in knowledge of effective and commercially viable methods to mitigate seabird bycatch during hauling in pelagic longline fisheries [22], and locally, to augment knowledge of causes and potential solutions to enable nearly eliminating seabird haul bycatch in this Hawaii fishery. Ca. 90% reductions in seabird bycatch have already been achieved in this fishery, and current fishing mortality levels are very unlikely to pose a risk to population viability or hinder plans for population rebuilding. Further reductions, however, would directly contribute, albeit slightly, to remediating cumulative effects from anthropogenic mortality sources, including removals in other pelagic and demersal longline fisheries operating in the north Pacific. Additionally, further reductions in seabird bycatch would improve fishing efficiency, as it is economically and operationally inefficient to catch, handle and release or discard seabirds. More importantly, identifying and implementing best practice methods to mitigate haul seabird bycatch augments the role of the Hawaii longline fisheries as a global model, potentially resulting in improved domestic and regional governance and fishing practices, catalyzing global seabird conservation gains.

## Methods

### PLOS ONE Community Standards for Data Availability

We analyzed observer program data for the Hawaii longline swordfish fishery. The Hawaii longline observer program dataset is subject to U.S. Government confidentiality restrictions under NOAA Administrative Order 216-100 on Protection of Confidential Fisheries Statistics. Third parties require authorization from the US National Marine Fisheries Service to obtain access to data from the Hawaii longline observer dataset; the dataset custodian provides public access to the confidential data if it will be used by researchers for analytical purposes subject to abiding by their non-disclosure standards.

### Study Period

We analyzed observer program data for the period beginning on 4 May 2004, when observers first began to conduct scan counts of seabirds, a regulation defining the gear design of a shallow, swordfish-targeting set went into effect, and 100% observer coverage began. The study period ended on 11 October 2012, the date of the most currently available record, based on the date and time of the start of the set.

### Proportion Hauled Alive

We determined the proportion of caught seabirds that were alive vs. dead upon gear hauling by calendar year and for the full study period, by individual and combined species. Observers record the condition of caught seabirds upon retrieval as being either alive not injured, alive injured, dead, or unknown [23]. The former two categories were used here as being retrieved alive. We used an odds ratio test using the epitools package for R [24], [25] to determine if there was a significant difference in the proportion of caught Laysan and black-footed albatrosses that were alive.

### Frequency of Live Seabird Captures per Set

The frequency of the number of live seabird capture events per set was determined.

### Proportion Seabirds Observed Caught During Haul vs. the Set and Condition

We reviewed comment fields for each seabird capture event to identify those that were observed being captured during hauling, were observed coming up on the gear from the gear soak (and assumed to have been captured during gear setting), or otherwise were not observed or recorded, and their condition (alive vs. dead) upon retrieval.

### Proportion of Live Seabirds Observed Caught by Branchline Placement and Tending

We reviewed observer comments for each seabird capture event to determine the proportion of live caught seabird that were observed being captured: (i) on a branchline still attached to the mainline, (ii) on tended branchlines, defined by NMFS [23] as branchlines that crew have unsnapped from the mainline and are being actively pulled in and coiled into a branchline bin; (iii) on untended lines, defined by NMFS [23] as branchlines that crew have unsnapped from the mainline and temporarily attached to the vessel with terminal tackle remaining in the water; (iv) entangled in the mainline but not entangled in a branchline or hooked; or otherwise (v) was not observed or recorded. During hauling, crew may use untended lines when they temporarily clip branchlines to the rail on the side or stern of the vessel when, for example, the gear becomes tangled or the crew coiling branchlines do not keep pace with hauling.

### Nominal Total and Live Seabird Catch Rate by Unique Vessel

We calculated nominal seabird catch rates for each unique vessel, including a nominal catch rate using the total number of observed seabird captures per 1000 hooks, and live seabird captures per 1000 hooks.

### Modeling Expected Live Seabird Catch

To determine the expected or mean catch of seabirds that were alive upon hauling, we fit observer data to a generalized additive nonparametric regression model with both fixed and random or mixed effects, referred to as a generalized additive mixed model (GAMM). This modeling approach: allowed for flexible specification of both the error and link functions, enabled arbitrary specification of the functional form for each continuous covariate included in the model, and accounted for mixed effects from multiple measurements on the same sampling unit of repeated longline trips by individual vessels [26]–[28].

The GAMM was fitted using: (i) thin plate regression splines to model nonlinear covariate effects except for any seasonal effect, where a cyclic cubic regression spline was used to reflect the cyclical seasonal behavior [28], (ii) a two-dimensional P-spline surface smoother to account for spatial effects attributable to the location (latitude, longitude) of each set, (iii) Poisson error structure appropriate for count (catch) data, (iv) log link consistent with use of count data modeled using Poisson error, (v) log offset for effort (hooks in a set) consistent with the log link and to account for modeling catch as a fixed proportional function of fishing effort, (vi) trip-specific heterogeneity as a random effect (random intercepts only) to account for the sampling structure of the data set, and (vii) all smoothness parameters in (i) and (ii) determined using generalized cross-validation [28]. These spatially explicit GAMMs are known as geoadditive GAMMs [29]. All the GAMM models were fitted using the *gamm4* package for R [30].

Because there is no accepted way to formally estimate model fit for GAMMs [28], [31], we implemented an approach used by Gilman et al. [13] of fitting an equivalent generalized additive model (GAM) to derive the percent deviance explained (a measure of GAM goodness-of-fit: see [26]), and to evaluate the importance of explicitly accounting for set-, trip-, or vessel-specific heterogeneity (the random effects attributable to the sampling design constraints) using a GAMM. We also explored the use of Tweedie GAMs and GAMMs to account for potential over-dispersion attributable to possible excess zero-catches for species, and employed the Tweedie family parameter = 1.19 based on Gilman et al. [13]. All Tweedie GAMs and GAMMs were fitted using the *mgcv* package for R [28].

The following covariates and factors were included in the standardized live seabird catch rate geoadditive GAMM (hereafter referred to as the “catch rate model”) due to their effects on catch rates of seabirds during pelagic longline hauling. Each variable is conditioned on the other 10 factors and covariates:

- **Year and month**: The year and month of fishing can affect nominal live seabird catch rates: There can be inter-annual and seasonal variability in the species composition of seabird assemblages and in seabird density at the fishing grounds, in absolute abundance of individual seabird species, in seabird scavenging behavior, in environmental variables that affect seabird efficacy at scavenging from fishing vessels, and in the spatial distribution of fishing effort [13]. E.g., Laysan and black-footed albatross density and intensity of scavenging behavior at the fishing grounds likely vary seasonally, as does the spatial distribution of fishing effort by Hawaii swordfish vessels [17], [32]. There can be seasonal segregation by age class at sea for Laysan and black-footed albatrosses: given that pre-breeding aged albatrosses may be more likely to be captured when scavenging from longline vessels relative to mature birds due to their inexperience, and may be more likely to scavenge from fishing vessels due to the trophic level of their diet [32]–[36], this presents another basis for expecting season to have a significant effect on nominal seabird catch rates.
- **Spatial location of fishing effort (vessel latitude and longitude at the start of hauling)**: Seabird species composition, density, age class, and foraging and scavenging behavior vary spatially [32]–[34], [36]. Thus, fishing vessel location can significantly affect the nominal live seabird catch rate.
- **Haul duration**: As both Laysan and black-footed albatrosses primarily forage during the daytime [37], and because Hawaii longline swordfish vessels conduct hauling predominantly during daylight hours, it is hypothesized that a longer hauling duration is positively correlated with the number of seabird captures.
- **Mean albatross density during hauling**: The number of Laysan and black-footed albatrosses present in an area around the fishing vessel during hauling significantly affects nominal catch rates [6], [38]–[40]. Every other hour during gear retrieval, from sunrise to 1 hour after sunset, observers estimate the number of individuals of each seabird species present within 137 m (150 yards) of the vessel, or otherwise record the absence of seabirds within this area at a point in time [23]. Observers make low, best and high estimates for each scan count. We used the medium/best category. Count estimates that used a general category ‘bird’ were excluded, as it was not possible to determine whether or not these included albatross species.
- **Mean of the Beaufort scale at the start and end of the haul**. The Beaufort wind force scale uses visual observations of the appearance of the sea surface (i.e., sea state) as an index for wind speed. Observers assign a numerical value, from force 0, when the sea surface is flat, to force 10, when there are very high waves >8.8 m (29 feet) [23]. Seabird agility while flying, and concomitant efficacy at scavenging from longline gear during hauling, is affected by wind strength [42], [43].
- **Weight of branchline swivel:** The weight of the swivel incorporated into branchlines, in combination with the distance of the swivel from the hook (next covariate), affect the depth of baited hooks during hauling [44]. This might affect the availability of baited hooks to Laysan and black-footed albatrosses when scavenging because these species are surface forages, and can reach baits only in the upper 1 m of the water column [35]. If more than one branchline weight amount is used on a vessel, then observers record the predominant weight amount [23]. This creates uncertainty in the weight amount employed for individual seabird catch events.
- **Distance of weighted swivel from the hook**: The leader length (distance between a weighted swivel and hook), in combination with swivel weight amount (previous covariate) affect the depth of baited hooks [44] and hence influence the ability of surface-scavenging Laysan and black-footed albatrosses to reach baited hooks during gear retrieval. Observers record leader and branchline lengths based on an average of a few random samples. There can be large variance in lengths in these gear components [44], which is not captured in the observer dataset.
- **Branchline length**. Branchline length may have a significant effect on seabird capture during hauling. The longer the branchline length, the longer time crew will take to retrieve the baited hook, and the higher the probability that the terminal tackle will be unprotected by the vessel hull at some point during hauling.
- **Records from vessels with disparate nominal live seabird catch rates**. Records are placed into three categories of sets having been made by 5 vessels with relatively low, 5 with relatively high, and 40 with average nominal live seabird catch rates. Vessels with nominal live seabird catch rates that are substantially different from the mean (as defined in Results section “*Nominal Total and Live Seabird Catch Rate by Unique Vessel*”) might employ gear designs and seabird haul mitigation methods that are significant in explaining the standardized catch rate for live seabirds.
- **Blue-dyed vs. untreated bait**: Dyeing fish bait blue can significantly reduce seabird catch rates in longline fisheries. This reduces the contrast between the bait and sea surface as viewed by seabirds when foraging from above [7], [11], [12]. Observers record whether bait is dyed blue upon gear hauling, where, “Properly dyed bait will be faded upon the haul back, but a light blue color will still be evident. If more than a few baits appear un-dyed or several un-dyed baits are on consecutive hooks (i.e., one or more baskets),” then observers record that baits were not dyed blue during hauling [23].

The model was fit to combined seabird species. Laysan albatrosses accounted for 73% of total live caught seabirds. The model, therefore, effectively identifies the significance of included factors on live Laysan albatross standardized catch rate. The sample size for live black-footed albatrosses was too small to produce a live black-footed albatross standardized catch model with high certainty. The covariate time of day of initiating hauling and factor offal and spent bait discarding practices during hauling were considered for inclusion in the model but rejected due to finding no significant effect on the seabird haul standardized catch rate and not improving model fit.

### Sample

We included in the study a subset of available records from the US National Marine Fisheries Service observer program dataset for shallow, swordfish-targeting sets made by the Hawaii longline fishery, as defined by [45] as having <15 hooks between floats, <20 m length float lines, 18/0 or larger 10° offset circle hooks, and mackerel-type bait. We excluded records that had not yet been validated and approved by the National Marine Fisheries Service at the time of running the query. We also excluded sets from designated research trips because experimental treatments may have affected fishing methods, gear and catch characteristics.

For study components that considered both captures that occurred during setting (likely to be retrieved dead) and hauling (likely to be retrieved alive), the sample included: (i) records of trips during which there were observations of one or more albatrosses present during observer bird scans during the set or during the haul, and/or (ii) one or more seabird was observed captured. Thus, records of trips in which there were no albatrosses observed attending the vessel during setting and hauling, and no seabirds captured, were excluded from the sample. These records were excluded because, in a fishing operation where no albatrosses were observed to be present during setting and hauling, the observation that no seabirds were captured is likely a result of an absence of albatrosses at the fishing grounds when gear was being deployed and retrieved, and not likely a reflection of seabird susceptibility to capture by that vessel [7].

Similarly, for the study component on the standardized catch model for live seabirds, which considered only captures that occurred during hauling, an additional 320 records where there were no albatrosses observed present during hauling were excluded. No seabirds, live or dead, were retrieved during these 320 sets. For hauls where there were no albatrosses observed present, the lack of live seabird captures was likely a result of the absence of albatrosses at the fishing grounds during hauling. We only considered the presence or absence of albatross species to determine whether to include a set in the analysis, as captures of other seabird species are very rare events in this fishery.

For the catch model, an additional 114 records were excluded from the sample for which observers did not record seabird scan count observations during hauling.

Finally, there were an additional 152 records removed from the sample used to fit to the standardized catch model due to missing data for one or more of the included factors or covariates.

Here seabird ‘captures’ are broadly defined to include observed and recorded: (i) pre-catch escapements, when seabirds were temporarily caught via hooking or entanglement but escaped prior to being landed onboard; (ii) pre-catch losses, when dead seabirds fell from the gear during hauling; and (iii) captures, when seabirds were caught in the gear and landed onboard.

## Results

Table 1 provides a summary of sample sizes included in the two study components: those evaluating both seabird captures that occurred during setting and hauling, and the live seabird catch rate model, which evaluated seabird captures during hauling.

**Table 1:** Summary of sample sizes for (a) study components considering both seabird captures during setting and hauling, and (b) a standardized catch model for live seabirds, which considered seabird captures during hauling.

Sample Size Parameter	Components Involving Both Captures During Set and Haul	Standardized Catch Rate Model for Live Seabirds
No. unique vessels	49	49
No. trips	679	665
No. sets/hauls	11,971	11,385
No. hooks	11,159,305	10,620,624
Total no. seabirds captured	517	481
No. live Laysan albatrosses captured	289	262
No. dead Laysan albatrosses captured	82	78
No. live black-footed albatrosses captured	99	96
No. dead black-footed albatrosses captured	45	43
No. live non-albatross seabirds captured^1^	2	2
No. dead non-albatross seabirds captured	0	0

^1^ One sooty shearwater, one Northern fulmar.

### Proportion Hauled Alive

**Figure 1:**
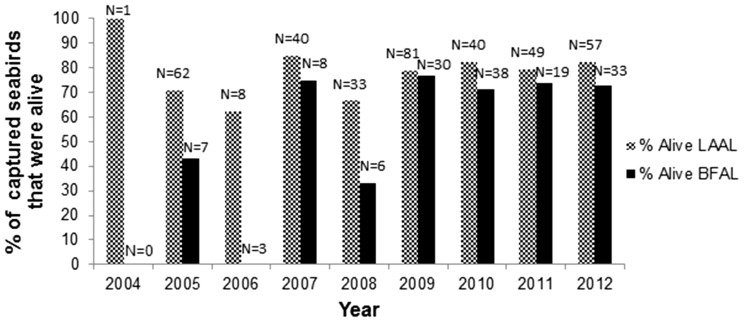
Percent of annual observed captures of Laysan (LAAL) and black-footed albatrosses (BFAL) that were alive at retrieval, Hawaii longline swordfish fishery, 2004–2012. Data labels are total number of LAAL or BFAL observed captured in that year.

Fig. 1 presents the proportion of total Laysan and black-footed albatross captures comprised of live birds by year, 2004–2012. Of 517 seabird captures, 75% were retrieved alive. The mean of nine annual percentages of total seabirds captured that were alive was also 75% (±5% SD of the mean). In each of the nine calendar years in the study period, a higher proportion of caught Laysan albatrosses were alive relative to black-footed albatrosses (Fig. 1). Over the study period, 78% of 371 retrieved Laysan albatrosses where alive, and 69% of 144 retrieved black-footed albatrosses were alive. Aggregated over the study period, Laysan albatrosses were 1.6 times more likely to be caught alive than black-footed albatrosses (odds ratio = 1.6, 95% CI: 1.01–2.51, P<0.05).

### Frequency of Number of Live Seabird Captures per Set

**Figure 2:**
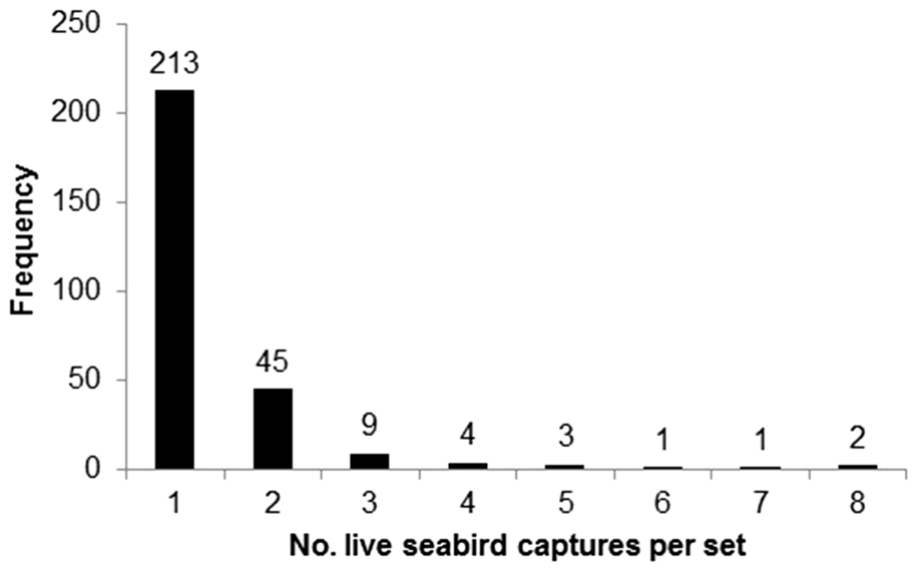
Frequency that the specified number of live seabird capture events occurred per set, Hawaii longline swordfish fishery, 2004–2012. Data labels are number of sets. Not shown, there were 11,693 sets with 0 seabird captures.

Fig. 2 presents the frequency that the specified number of live seabird capture events occurred per set. Of 278 sets with one or more live seabird capture, 77% (213) had a single live seabird capture.

### Proportion of Seabirds Observed Caught During the Haul vs. the Set and Condition

There were 230 records of seabird captures that included information on whether the bird was captured during the haul or otherwise came up on the gear from the soak, and included information on the condition of the seabird upon retrieval. Of these, there were 222 records of seabirds retrieved alive, all of which were observed as having been caught during hauling. Of the 8 records of seabirds retrieved dead, all were observed as having come up on the gear from the soak (inferred to have been caught during the set, or less likely due to the diving abilities of the seabirds that interact with the fishery, during the gear soak).

### Proportion of Live Seabirds Observed Caught by Branchline Placement and Tending

Of 222 live capture events observed to have occurred during hauling, observers recorded the manner of capture for 165 records. Of these, 2 (1.2%) were observed becoming entangled in the mainline (but not also hooked or entangled in a branchline), 4 (2.4%) captured on branchlines attached to the mainline, 134 (81.2%) captured on tended branchlines, and 25 (15.2%) captured on untended branchlines.

There were extremely small sample sizes of records with information on the manner of seabird haul captures for the initial half of the time series: the average annual number of records with this information was 4 for 2004 to 2008, and 37 for 2009 to 2012. The proportion of haul captures on untended lines was not significantly explained by year (i.e., there was no significant temporal linear trend) based on fitting the data series to a simple linear regression model, with poor model fit (p>0.05, R^2^ = 0.02).

### Nominal Total and Live Seabird Catch Rate by Unique Vessel

Nominal live seabird catch per unit of fishing effort (CPUE) ranged from 0 to 0.17 with an average of 0.028 live birds/1000 hooks ±0.006 SD of the mean, N = 49. Nominal total (live and dead) seabird CPUE ranged from 0 to 0.21 with an average of 0.037 birds/1000 hooks ±0.007 SD of the mean, N = 49.

**Figure 3:**
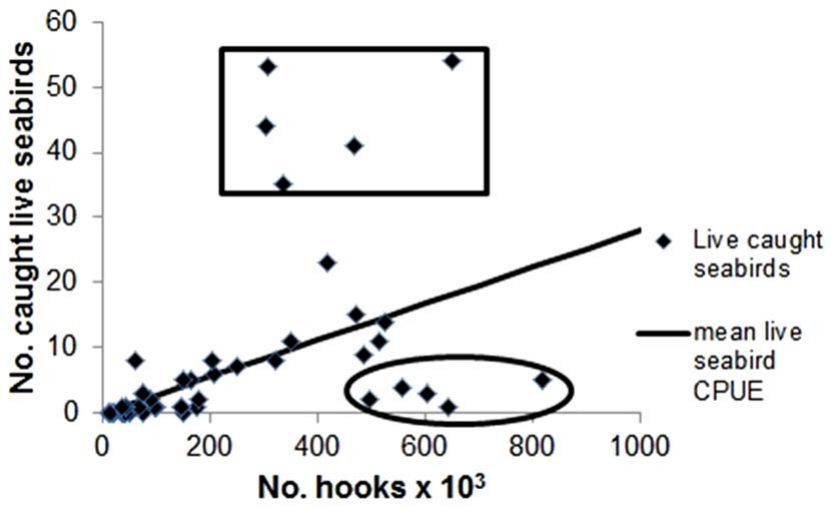
Number of live seabirds observed captured per number of shallow-set hooks set, for 49 Hawaii longline swordfish vessels, 4 May 2004 - 11 October 2012. Solid line is mean live seabird CPUE of 0.028 live birds per 1000 hooks. Outliers with relatively high and low nominal catch rates for live seabirds are identified within the rectangle and oval, respectively, per rules defined in Results section “*Nominal Total and Live Seabird Catch Rate by Unique Vessel*”.

Fig. 3 is a plot of nominal live seabird CPUEs by vessel. Of the 49 vessels included in the study, for vessels that set >300,000 hooks during the study period, there were five low outliers with nominal live seabird CPUEs that were below the mean by more than 3.5 SD of the mean (<0.007 live birds/1000 hooks). There were five high outlier vessels that set >300,000 hooks with nominal live seabird CPUEs that exceeded the mean by more than 5 SD of the mean (>0.058 live birds/1000 hooks).

Of the factors and covariates found to have a significant effect on the standardized haul seabird catch rate, the five outlier vessels with high seabird haul catch rates had significantly higher means for year, month, albatross density, swivel weight amount, leader length, and branchline length, and significantly lower mean Beaufort scale than the vessels with average live seabird catch rates (p<0.01, Student's two-tailed paired t-test, for all 7 variables). The five outlier vessels with low catch rates had significantly higher means for year, month, haul duration, swivel weight amount, and leader length, and significantly lower mean albatross density and branchline length than the fleet average (p<0.01, Student's two-tailed paired t-test, for all 7 variables). There was no significant difference in means between high outlier and fleet average haul duration, and between low outlier and fleet average Beaufort scale (p>0.05, Student's two-tailed paired t-test).

### Standardized Catch Model for Live Seabirds

Table 2 presents Akaike Information Criterion (AIC) values for the GAMM and equivalent GAM, and results of a log-likelihood ratio test [28]. A smaller comparative AIC value indicates a relatively better fitting model, and the formal log-likelihood ratio test determines if the difference in deviance between the GAMM (linear mixed effects regression) and GAM (linear regression) models was significant. The GAMM was a significantly better fitting model than the equivalent GAM, which did not account for set-, trip- or vessel-specific heterogeneity. The GAMM catch model would explain more than the 44% of variance explained by the GAM-equivalent model (Table 2).

**Table 2:** GAMM and GAM-equivalent standardized catch rate model for live seabirds fits to the Hawaii longline swordfish fishery observer program dataset.

AIC		Log-likelihood Ratio Test			% Variance
GAMM	GAM	LLR Value	df	P	Accounted for by GAM
13,369.2	13,799.3	432.2	1	<0.0001	44

**Figure 4:**
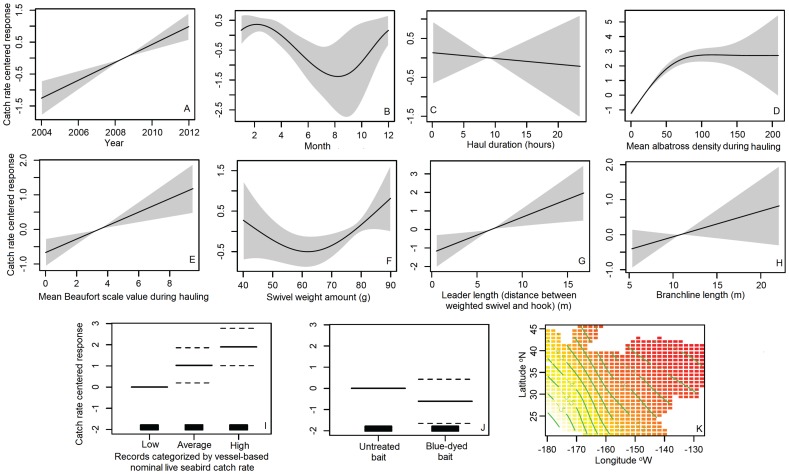
Generalized additive mixed Poisson regression model (GAMM) fitted to live seabird catch in hauls made by the Hawaii longline swordfish fishery (N = 11,385 hauls), 4 May 2004 - 11 October 2012. The GAMM explicitly accounts for the effects on the standardized catch rate model for live seabirds of: (A) year, (B) month, (C) haul duration, (D) mean albatross density during hauling, (E) mean value of the Beaufort scale during hauling, (F) amount of the weighted swivel on the branchline, (G) distance between the weighted swivel and hook, (H) branchline length, (I) vessels with low, average, or high nominal live seabird catch rates, (J) untreated vs. blue-dyed fish bait, and (K) two-dimensional spatial (setting location).

The catch rate GAMM was an adequate fit to the dataset, with both significant nonlinear effects and no apparent aberrant residual behavior (Fig. 4). Fig. 4 presents the GAMM fit to the live seabird catch data conditioned on nine covariates (year, month, location at the start of the haul, mean albatross density during hauling, haul duration, mean Beaufort scale value, branchline weight amount, distance of weighted swivel from the hook, and branchline length) and two factors (hauls categorized as having been made by vessels with high, low and average nominal live seabird catch rates, and blue dyed vs. untreated bait).

Each panel of the GAMM plot in Fig. 4 is on the same y-axis scale, allowing for the identification of the relative contribution of each covariate and factor in explaining model variability. In all but the final panel, the response is shown centered on the y-axis scale. In panels for covariates (panels A–H), solid curves are the model fit, and the shaded area is 95% pointwise confidence bands. In panels for factors (panels I and J), solid bars are the mean, dashed bars are the 95% confidence interval, and the first factor is the reference level, which is centered at zero on the y-axis.

Covariates year, mean Beaufort scale value, and distance between the weight and hook were significant linear effects in the model, while haul duration and branchline length were not significant effects (Fig. 4A, C, E, G, H). Over the study period, there was a strong increasing trend in the standardized catch rate for live seabirds (Fig. 4A). Standardized catch rates were significantly higher the larger the Beaufort scale value, and distance of the weight from the hook (Fig. 4E, G). Covariates month, mean albatross density during hauling, and swivel weight amount were significant nonlinear effects (Fig. 4B, D, F). Standardized catch rates were highest in winter months, when fishing effort is highest (hence the relatively narrow confidence bands), and lowest in late summer/early autumn (Fig. 4B). The standardized catch rate increased steeply as mean albatross density around the vessel increased to about 75 individuals, and then leveled off, but the sample size diminished substantially after about a density of 125 albatrosses within the area 137 m from the vessel (Fig. 4D). The standardized catch rate significantly increased as the weight of swivels increased from 65 g. There was no significant effect on the standardized catch rate as swivel weight increased from 40 g to about 65 g (Fig. 4F). There were significantly higher standardized catch rates in hauls made by vessels with relatively low nominal live seabird catch rates relative to both those with average and high nominal catch rates, this being a relatively small effect, and there was no significant difference in standardized catch rates between hauls made by vessels with average vs. high nominal live seabird catch rates (Fig. 4I). There was no significant effect on the standardized catch rate for live seabirds between hauls made with vs. without blue-dyed bait (Fig. 4J). Relatively lower standardized catch rates occurred when moving northeast from the main Hawaiian Islands, and higher rates occurred when moving westward from the main Hawaiian Islands (Honolulu in the main Hawaiian Islands is located at about 21°N, 158°W) (Fig. 4K). Of the covariates and factors included in the model, mean albatross density during hauling, distance between the weight and hook (leader length), and year had the largest contributions in explaining model variability, in the order listed, with smaller, about equivalent effects for the remaining significant covariates and factor (Fig. 4).

## Discussion

### Proportion Caught Alive and Condition of Birds Observed Caught on the Haul vs. Set

Of the records where information was available on both whether a bird was captured during hauling or came up on the gear from the soak and the condition of the bird upon retrieval, all 222 seabirds recorded as retrieved alive were haul captured, and all 8 landed dead came up from the soak. This supports the hypothesis that seabirds retrieved alive were caught during hauling, and that seabirds caught during the deployment of shallow-set gear have a low probability of surviving the gear soak and will be dead upon gear retrieval. Furthermore, over the study period, an average of 75% of seabirds retrieved during hauling were alive. This suggests that since seabird regulations came into effect, most seabird captures in the Hawaii longline swordfish fishery occur during hauling, and not during setting. Any additional efforts to reduce seabird bycatch should therefore focus on reducing interactions during hauling.

### Proportion Hauled Alive by Species

A significantly smaller proportion of black-footed albatrosses are retrieved alive relative to Laysan albatrosses. This suggests that, while these two sympatric north Pacific albatross species primarily forage during the daytime [21], [37], relative to Laysans, black-footed albatrosses might be a more active nighttime scavenger, overlapping with the time of day of gear setting. In addition, the relatively less-abundant and bulkier black-footed albatrosses may be less competitive at scavenging from fishing vessels during the daytime than Laysan albatrosses [35]. The finding indicates that mitigating haul bycatch would be a larger benefit to Laysan albatrosses.

### Frequency of Number of Live Seabird Captures per Set

The majority (77%) of live bird captures occurred as single events (a single live bird capture during an individual haul) (Fig. 2). Haul seabird captures are therefore a rare event in that they are not typically occurring as multiple captures in a single haul, for example, as a result of crew deploying a large number of untended lines. Additional mitigation methods would therefore require continuous implementation to reduce the risk of haul seabird captures when albatrosses are present during hauling, and not a change in fishing practices or gear only after an initial live bird capture occurs.

### Proportion of Live Seabirds Observed Caught by Branchline Placement and Tending

Given that most (81%) of haul-caught seabirds are on tended lines, mitigation methods during hauling should consider how best to reduce the risk of bird access to terminal tackle as crew coil branchlines into bins. The small proportion of observed haul seabird captures on untended lines (15%) suggests that few branchlines are untended, and that they are untended only briefly. The small number of observations of birds being captured during hauling on branchlines still attached to the mainline, and entanglements in the mainline suggests either that terminal tackle generally does not drag on the sea surface for branchlines attached to the mainline during hauling, and/or that these capture events generally occur beyond observers' range of detection.

### Standardized Catch Model for Live Seabirds

Results from the AIC and log-likelihood test support the inference that the GAMM would account for more of the deviance than the equivalent GAM (Table 2), indicating that inclusion of random effects in the model was an improvement [13].

Of the covariates and factors included in the model, the three with the largest contributions in explaining model variability were mean number of albatrosses within 137 m of the vessel during hauling, leader length and year, in that order (Fig. 4).

There was a significant linear increasing trend in the catch model over the time series. For comparison, the nominal live seabird catch rate doubled from the first three years of the data series (0.02 live seabirds/1000 hooks) to the latter three years (0.04 live seabirds/1000 hooks). The increasing seabird haul catch rate may partly be due to an increasing trend in the mean number of albatrosses attending vessels during hauling: The mean number of albatrosses attending vessels during hauling was significantly explained (i.e., was temporarily confounded) by year, increasing at a rate of 1 albatross within 137 m of the vessel during hauling per year when the series is fit to a simple linear regression model (p<0.05, R^2^ = 0.46). This may reflect trends in absolute abundance, increased scavenging from fishing vessels perhaps due to decreasing availability of natural prey (e.g., due to reduced relative abundance of tunas, which reduces the availability of baitfish to pelagic seabirds), and/or increased breeding activity during the study period [16,.46].

**Figure 5:**
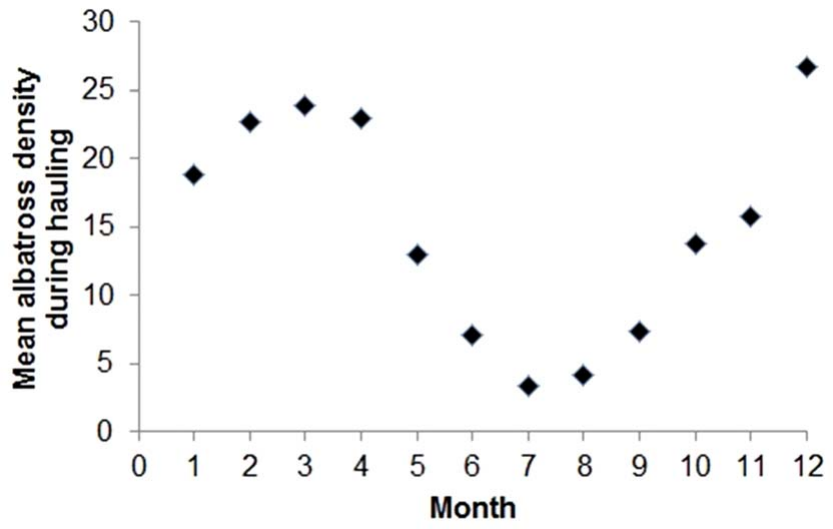
Mean albatross density (mean number of Laysan and black-footed albatrosses within 137 m of the fishing vessel) during hauling by month, Hawaii longline swordfish fishery, May, 2004 - October, 2012.

The effect of season in the model is likely linked to the monthly variability of albatross density at the fishing grounds: relative (local) abundance of Laysan and black-footed albatrosses at the Hawaii longline swordfish fishery grounds was found to be highly variable by month, with highest densities from December to April (mean of 23.0±1.3 SD of the mean), and lowest from May to November (mean of 10.9±1.9 SD of the mean) (Fig. 5). The significant effect of month on the live seabird catch rate, with relatively highest standardized catch rates from January to March (Fig. 4B), corresponds with periods when mature Laysan and black-footed albatrosses are brooding and beginning to rear chicks. At this stage, they make relatively short foraging excursions (short excursions during the brooding period, and mixing short and long trips during the rearing period) into areas that overlap substantially with the distribution of the Hawaii longline swordfish fishery grounds from their breeding colonies in the Northwestern Hawaiian Islands in order to provide a frequent rate of chick-feeding when one parent is continuously tending the chick [32], [47]. The lowest live seabird catch rates occurred in August and September during the nonbreeding season for these North Pacific albatross species, when mature individuals have recovered from the energetic demands of the breeding season, and forage in areas that overlap less with the fishing grounds of the Hawaii longline swordfish fishery relative to the distribution of breeders during the breeding season [21], [32], [47]. There was low dispersion in the seasonal distribution of fishing effort (quarter 1 mean of 50.0%±8.9 SD of the mean, quarter 2 mean of 28.1±6.0 SD of the mean, quarter 3 mean of 2.7±0.7 SD of the mean, quarter 4 mean of 19.2±10.4 SD of the mean). The distribution of fishing effort by season was not significantly explained (i.e., confounded temporarily) by year when the series was fit to a simple linear regression model (p>0.05 for each quarter, R^2^ = 0.08, 0.02, 0.21, 0.20, for the four quarters, respectively, 2005–2011).

The small variability in mean haul duration during the study period, with a mean of 8 h 57 min±6.4 min SD of the mean, might explain the observed lack of significant effect in the model of the covariate haul duration. It was hypothesized that a longer time spent retrieving gear provides a longer opportunity for seabirds to interact with baited hooks and get caught.

The standardized catch rate increased steeply as mean number of albatrosses around the vessel increased to about 75 individuals. This confirms previous findings of the positive relationship between seabird catch rates and the number of seabirds scavenging from a fishing vessel [6], [38]–[41].

The observation of a significantly higher standardized catch rate with higher wind strength (Beaufort scale) is consistent with previous studies, and with the understanding that albatrosses have improved agility and scavenging ability with higher wind strength [42], [43]. There was relatively low dispersion in mean Beaufort scale value during hauling during the study period (±0.07 SD of the mean) and the variable was not significantly explained by year when the data series was fit to a simple linear regression model (p>0.05, R^2^ = 0.3). Wind direction in relation to the vessel hauling direction is another potentially significant explanatory variable [42], [43] not explored in this study due to the unavailability of this variable in the observer program dataset [23].

There was an observed significant effect of an increase in swivel weight above 65 g in reducing the standardized catch rate for live seabirds, but no significant effect of weights between 40 g and 65 g. These observations are inconsistent with the hypothesis that, during hauling, baited hooks are more likely to remain below the reach of scavenging albatrosses the larger the swivel weight, if the weight is sufficiently close to the hook. However, most records had weights far from the hook (discussed below), likely reducing the effect of swivel weight amount on the depth of hooks during hauling. There was low variability in swivel weight, with a mean of annual means of 72.4 g±0.97 SD of the mean, where 64% of records had an 80 g swivel. Swivel weight was not significantly explained by year when the data series was fit to a simple linear regression model (p>0.05, R^2^ = 0.008).

The finding of a significantly higher catch rate with longer leaders (the distance between a weighted swivel and hook) suggests that during hauling, baited hooks are more likely to remain below the reach of scavenging albatrosses the closer the swivel is to the hook. This effect is relevant both to tended (actively being coiled) and untended (temporarily not being coiled) branchlines. There was low variability in this variable, with a mean of annual means of 6.6 m±0.12 SD of the mean for leader length. Of the 11,385 records, only 403 (3.5%) placed weighted swivels within 1 m of the hook, which is required when opting to side set [19], discussed in more detail later. Leader length was significantly explained (i.e., was temporarily confounded) by year, but only decreasing by 0.09 m per year, when the series was fit to a simple linear regression model (p<0.05; R^2^ = 0.48).

The small variability in branchline length with a mean of annual means of 10.9 m±0.15 SD of the mean might explain the observed lack of significant effect of this covariate in the catch rate model. It was hypothesized that shorter branchlines result in a lower likelihood of seabird capture during hauling because they require less time for crew to coil and are less likely to be unprotected by the vessel hull during coiling or when unattended. However, shallower setting via the use of shorter branchlines could exacerbate problematic bycatch of other species groups, including sea turtles and pelagic sharks [10]. Branchline length was not significantly explained by year and a poor, highly uncertain fit to a simple linear regression model (p>0.05; R^2^ = 0.01).

The existence of the outlier vessels, with nominal live seabird catch rates that were substantially different from the mean, might be partly explained by differences in average albatross density during hauling. There was a significantly higher hauling albatross density for the five high outlier vessel, and significantly lower density for the five low outliers relative to vessels with average catch rates, and the number of albatrosses attending vessels during hauling was found to have the highest effect in the standardized catch model. Other factors, such as crew hauling methods, including rate of coiling branchlines and frequency of terminal tackle dragging astern, are other possible significant explanatory variables.

The lack of a significant effect on the standardized catch rate between hauls made with vs. without blue-dyed bait suggests that dyeing fish bait a darker blue color does little to reduce the ability of seabirds to detect them during hauling after a ca. 9 hour-long gear soak, and hence to significantly affect the seabird standardized catch rate during daytime hauling. The small sample size of hauls with untreated bait (6%, 691 of 11,385) may have affected the model account of this factor. Blue-dyed fish bait, in some cases used in combination with other seabird bycatch gear technology mitigation methods, has been found to significantly reduce seabird bycatch rates, but blue-dyed fish bait has been found to be less effective than blue-dyed squid bait at avoiding seabird interactions [7], [11], [12], [48]. Studies have not demonstrated a significant effect of blue-dyed bait on sea turtle catch rates relative to untreated bait [49].

The observation of relatively lower standardized catch rates when moving east and northeast from the Laysan and black-footed albatross breeding colonies in the Northwestern Hawaiian Islands is consistent with documentation of breeders making short foraging excursions during the brooding period [21], [32], [47]. This corresponds to the period when the Hawaii longline swordfish fishery conducts the majority of effort (>50% of hooks are set during the first quarter of the year).

Several variables not included in the standardized catch model that may have improved model fit were considered and not included due to data quality issues, inclusion of the variable did not improve model fit, or the variable was not available in the observer dataset. Gilman et al. [13] reviewed factors that may have a significant effect on catch rates, a subset of which are of relevance to catching seabirds during longline hauling. For example, hook size and design, and bait type, which might have an effect on seabird captures [10], [13], were not included in the model. The swordfish fishery was required to use 18/0 circle hooks with a 0°–10° offset, and mackerel-type fish bait during the entire study period. Fish species used for bait might significantly affect the live seabird catch rate, for instance, if different species of fish bait are substantially different in texture and concomitant difficulty required for seabirds to bite off pieces and to remove them from a hook. However, 96.8% of sets employed Atlantic mackerel (saba) (*Scomber scombrus*) for bait, with very small sample sizes for sets employing other bait types.

A factor for the two alternative regulatory-defined combinations of seabird bycatch mitigation methods [19], [20] was not included in the model. Only 3% (386)of sets included in the sample for the standardized catch rate model were recorded by observers as having conducted side setting. In most observer records of vessels recorded as “side setting”, however, the vessel was not employing all regulatory-required elements. Therefore using the individual gear technology methods as model variables that are understood to affect seabird haul captures that are included as part of the regulatory definition of side- vs. stern-setting is more rigorous than using the two regulatory seabird bycatch mitigation categories as a model factor. The standardized catch rate model explicitly accounted for some of the regulation variables likely to significantly affect the seabird haul captures of swivel weight amount, leader length, and blue-dyed vs. untreated bait, the latter being observed to not have a significant effect. We also explored the effect of discard practices during hauling and time of day of initiating hauling, both found not to have a significant effect, discussed in more detail below. The two other factors associated with side vs. stern setting defined in the seabird regulations of using a bird curtain during side setting and location on deck of setting the main line [19], [20] likely have no effect on seabird interactions during hauling. Findings here suggest that side-setting vessels likely have lower seabird haul capture rates than stern-setting vessels due to the side-setting vessels being required to place weights close to hooks. Given that almost all vessels conventionally use swivel weights >45 g (only 15 of 11,385 records had swivel weights <45 g), the minimum weight requirement included as part of the side setting regulatory definition would not have an effect. It is unclear, however, whether daytime side-setting vessels would have higher or lower seabird catch rates during setting relative to vessels setting from the stern at night.

The time of day of initiating hauling, when included as a covariate in the catch model, did not have a significant effect and did not improve model fit. This was considered a potentially significant explanatory variable on live seabird captures based on observations that Laysan and black-footed albatrosses are primarily diurnal foragers [21], [37]. However, >99% of hauls included in the study sample overlapped morning hours: the mean time of day of initiating hauling was 6∶51 with low dispersion (±0.5 min SD of the mean).

When included as a factor in the model, records where crew discarded offal and spent bait on the opposite side of the vessel from the hauling station did not have a significantly different standardized catch rate for live seabirds from records when these discards were not made on the opposite side of the vessel, and inclusion of this factor did not improve model fit. Observers record whether or not crew discarded offal and spent bait on the opposite side of the vessel from the hauling station [23], which might draw seabirds attending the vessel away from the hauling station, reducing the probability of interactions with gear, but might also increase the density and foraging intensity of seabirds relative to vessels that refrain from discharging any biomass during hauling [10]. Refraining from discharging offal, spent bait, dead discards, and live catch may be more effective at reducing seabird bycatch over the long-term [9], [10]. There was a small sample size for hauls where there was no discarding of offal and spent bait on the opposite side of the vessel from the hauling station: Of 11,385 records included in the sample, <1% (111) of hauls had neither offal nor spent bait discarded on the opposite side of the vessel during hauling.

There could be a vessel effect on the live seabird catch rate due to unique attributes of the vessel, gear design, and methods employed by the operator and crew that are employed somewhat consistently across trips and sets made by that vessel [13]. And attributes of a trip affect the catch during constituent sets, i.e. the sets in a trip are potentially correlated, for example, through oceanographic and environmental conditions and aspects of the fishing gear, vessel and fishing methods unique to that trip [13], [50]. In this study, trip was used instead of unique vessel or set as a random effect in the model as this was found to result in a better model fit, and because the use of trip as the model random effect accounted for both the effect of constituent sets in a unique trip, and effect of each unique vessel which made a subset of the trips included in the study sample.

### Estimating ‘Cryptic’ Sources of Seabird Fishing Mortality

Here seabird ‘captures’ included observed and recorded: (i) pre-catch escapements when seabirds were temporarily caught via hooking or entanglement but escaped prior to being landed onboard; (ii) pre-catch losses when dead seabirds fell from the gear during hauling (but not seabirds that were caught during setting and were removed from the gear during the soak); and (iii) captures, when seabirds were caught in the gear and landed onboard. Several other potential sources of fishing mortality from fishing operations were not estimated or accounted for in this study, resulting from cryptic, largely undetectable losses from pre-catch, post-release, ghost fishing, collateral effects, cumulative interactions, and synergistic effects [46]. These other sources of fishing mortality can be substantial. For instance, findings from experiments in the Hawaii longline fisheries have estimated that seabird pre-catch losses are about 50% of the total seabirds observed hauled aboard (i.e., about a third of the seabird caught during setting are not hauled aboard) [12], [41], [51]. Furthermore, an unknown proportion of seabirds that escape and are released alive will survive [46]. There are also indirect, collateral effects from direct seabird fishing mortalities. For example, the removal of one albatross of a breeding pair from fisheries capture typically results in chick mortality by starvation, and the remaining albatross will take several years before mating again, further reducing reproductive output [46], [52].

### Key Findings and Next Steps

Through the development of a standardized catch rate model for live, haul-caught seabirds, this study has identified opportunities to nearly eliminate seabird bycatch in the Hawaii longline swordfish fishery. Globally, study findings fill a gap in knowledge of gear technology methods to mitigate seabird bycatch during pelagic longline hauling. The study has identified opportunities to avoid and minimize seabird capture during hauling through changes in fishing gear and hauling methods, including through using shorter leaders and heavier swivels. Most haul captures were on branchlines actively being retrieved, indicating that haul mitigation methods should consider how best to reduce the risk of bird access to terminal tackle as crew coil branchlines. The study has also shown that a higher density of albatrosses attending vessels during hauling results in higher seabird haul catch. The proportion of Laysan haul captures were significantly higher than for black-footed albatrosses, perhaps due to differences in the time of day of foraging and in daytime scavenging competitiveness; this suggests that mitigating haul bycatch would be a larger benefit to Laysans.

Locally, future research priorities are to assess the commercial viability of the factors determined here to have a significant effect on seabird haul capture. Furthermore, other factors that have the potential to significant affect seabird haul captures that are not available in the observer program database, including faster branchline coiling and equipment that deters seabirds from entering the area where terminal tackle becomes accessible, should also be assessed for efficacy and commercially viable.

A commercial demonstration of promising gear technology methods to reduce seabird haul bycatch would help confirm their efficacy, determine their economic viability, safety and practicality, and develop industry support for uptake of effective mitigation methods determined to be commercially viable. A commercial demonstration could provide free equipment and cover any installation costs to swordfish vessel owners who volunteer to participate in the trial, found to be effective in gaining voluntary participation in a trial of side setting in the Hawaii longline fishery [44].

Given the finding that most haul bycatch was observed to occur on tended lines, trials should focus on methods with promise to effectively reduce seabird captures on branchlines as they are being coiled. This includes methods identified here of shorter leaders and heavier swivels. Other potentially effective methods include optimizing coiling rates, preventing or reducing the incidence of terminal tackle dragging at the sea surface far from the protection of the vessel hull, and deterring birds from entering the area where terminal tackle comes to the surface during hauling.

Reduced leader length and increased swivel weight were variables in the standardized haul catch rate model with significant effects that would be effective in reducing seabird captures on tended as well as untended lines. Shorter leaders place weighted swivels closer to baited hooks, possibly reducing the probability and time that terminal tackle reaches the surface during hauling. Heavier branchline weights when attached close to the hook can keep baited hooks sufficiently below the surface during hauling so that they cannot be detected or otherwise are out of reach of Laysan and black-footed albatrosses. However, placing weights close to hooks on branchlines lacking wire leaders may increase safety risks: if a branchline breaks during hauling, which frequently occurs when sharks are caught and bite off the terminal tackle, or if the hooks pulls free from a caught fish with the line under high tension (the fish ‘throws’ the hook), the weight can fly at the vessel at high velocity, posing a safety risk to crew [10], [53]. Using wire leaders when weights are placed closed to hooks, or using new designs for weights that reduce the safety risk to crew [10], [54] are possible solutions. However, because significantly lower shark catch rates occur with monofilament leaders vs. leaders of more durable material (wire, multifilament nylon) because sharks can bite through the monofilament [55], using wire leaders with weights close to the hook could reduce seabird haul catch but at a cost to sharks. Therefore, there is a need to assess the relative risks to populations and stocks subject to fishing mortality from alternative bycatch mitigation gear technology methods, including leader material and branchline length (discussed earlier).

Observers noted several causes of baited hooks of tended and untended branchlines to trail behind the vessel, including: (i) temporarily stopping mainline hauling and/or stopping the vessel, e.g., to retrieve buoy lines and radio beacons, land a fish, bring a shark close enough to the vessel hull to allow crew to cut the leader and release it, disentangle gear, and lower fish into the hold; (ii) when branchlines tangle and hooks get caught on the mainline; (iii) relatively slow branchline coiling, causing baited hooks to slowly skip across the sea surface; and (iv) when crew cannot keep up with coiling branchlines at the rate that they are coming up on the mainline, resulting in crew temporarily attaching branchlines to the vessel, typically attached at the stern-side of the hauling station. In combination with branchline length, leader length, and swivel weight, the position on deck where crew clip untended branchlines to the vessel, and the position of crew when coiling branchlines into totes likely also determine whether terminal tackle drags astern when one of these four scenarios occurs. If these two positions could be located at a distance forward from the stern that exceeds the length of branchlines, then this could reduce the incidence of terminal tackle dragging astern during hauling.

Seabirds could be prevented from accessing the area where baited hooks come to the surface through deployment of various deterrents, such as a towed buoy, bird curtain and tori line [9]. About half (24) of vessels voluntarily used a towed buoy during 757 (6%) of the hauls made during the study period, and 2 vessels voluntarily used a tori line during 5 (0.04%) of hauls. A bird curtain could employ the same design as developed for use during side setting on pelagic longline vessels [12], [19], [44] with an aim to keep seabirds from entering the area where baited hooks are near and at the sea surface during hauling. The bird curtain could be situated perpendicular from the vessel on the side where hauling occurs so as to prevent scavenging seabirds from being able to establish a flight pattern that brings them close to the vessel hull where terminal tackle is accessible at the sea surface during branchline hauling. Demersal longline vessels in some fisheries use a ‘Brickle’ curtain design that is a bird curtain positioned parallel to the vessel hull in front of the hauling station, to avoid seabird interactions during gear hauling, by preventing birds from flying into the area where the line is being hauled, and preventing birds that are sitting on the surface from swimming into the hauling bay area [56], [57]. The rectangular-shaped brickle curtain used on demersal longliners is unlikely to be effective for protecting pelagic longline branchlines during hauling because pelagic vessel branchlines are much longer than demersal longline snoods, causing pelagic longline baited hooks to be available to birds over a much larger distance from the vessel relative to demersal vessels.

Discussed previously, while conflicting with the current Hawaii seabird regulations [19], refraining from discharging offal, spent bait, dead discards, and live catch during hauling, or during all fishing operations, may result in lower seabird bycatch over the long-term relative to vessels that discard material away from the area where gear is being deployed or retrieved [9], [10]. Vessels that routinely discard material may increase the density and foraging intensity of seabirds relative to vessels that refrain from discharging.

An automatic electric branchline coiler (known as snood pullers for demersal longline vessels) [58] would have the potential to reduce the time required for crew to retrieve branchlines relative to manual retrieval, and hence reduce the time that baited hooks are available to scavenging seabirds. Automatic coilers were historically used in the Hawaii longline fishery when traditional basket-style gear with tarred rope was used, before transitioning to monofilament gear. With the modern gear, manual coiling into bins may be more efficient and be less likely to result in branchline tangles during setting than using automatic coilers (Jim Cook, Hawaii Longline Association, personal communication, 15 Nov. 2012).
